# Intelligent system for human activity recognition in IoT environment

**DOI:** 10.1007/s40747-021-00508-5

**Published:** 2021-09-07

**Authors:** Hassan Khaled, Osama Abu-Elnasr, Samir Elmougy, A. S. Tolba

**Affiliations:** Computer Science Department, Faculty of Computers and Information, Mansoura University, Mansoura, Egypt

**Keywords:** IoT, Human activity recognition, Capsule neural network, Intelligent system, Decision support system

## Abstract

In recent years, the adoption of machine learning has grown steadily in different fields affecting the day-to-day decisions of individuals. This paper presents an intelligent system for recognizing human’s daily activities in a complex IoT environment. An enhanced model of capsule neural network called 1D-HARCapsNe is proposed. This proposed model consists of convolution layer, primary capsule layer, activity capsules flat layer and output layer. It is validated using WISDM dataset collected via smart devices and normalized using the random-SMOTE algorithm to handle the imbalanced behavior of the dataset. The experimental results indicate the potential and strengths of the proposed 1D-HARCapsNet that achieved enhanced performance with an accuracy of 98.67%, precision of 98.66%, recall of 98.67%, and F1-measure of 0.987 which shows major performance enhancement compared to the Conventional CapsNet (accuracy 90.11%, precision 91.88%, recall 89.94%, and F1-measure 0.93).

## Introduction

An Intelligent Decision Support System (IDSS) is an ideal approach for solving many challenges that can currently face the world. With the popularity and widespread of Machine Learning (ML) algorithms, the creation process of IDSS is easier and faster combined with the easy access to big datasets covering all aspects of our life which helped to fight COVID-19 virus [1]. IDSS helps physicians in detecting the virus in an early stage which increases the probability of survival of the patients. Moreover, recognizing the patients’ hand gestures is a popular application of IDSS in the field of smart healthcare systems. It alerts the staff for the patients’ requests in time without delays in remote monitoring environments [2]. The importance of IDSS in the medical fields is especially appreciated in poor countries as the healthcare service is very weak and, in some places, it does not exist. IDSS can fill the gaps in the services by providing on-time and cheap service without the need for expensive equipment and trained personnel.

Nowadays, there is tremendous growth in IoT-enabled devices for empowering decision-making processes in complex systems. The fast development and miniaturization of sensors and reduced need for power requirement leads to a revolution in the field of Human Activity Recognition (HAR). Detection of early signs of critical disease like diabetes [3] and heart disease [4], even detection of early signs of COVD-19 using smart watches’ [5] sensors data, became a reality.

One trend that has gained importance recently is moving from one size fits all in the field of medicine to Personalized Health Care (PHC) and medicine [6, 7]. This happened due to the growth in aging population and the rise of the costs of chronic diseases. Therefore, a new solution is needed for this problem. This solution should include new ways to monitor and measure the vital signs of every patient to tailor and customize the medication plan for specific needs. This can be achieved through using ML and the Internet of Things (IoT) through using suitable sensors around the patient which send the data continuously to the doctors and hospitals to make informed decisions. Such information is used to help the beneficiaries regarding controlling the daily-life activities [8].

The idea behind this paper is to develop an IDDS for automatically collecting and classifying the daily-life activities through integrating the power of IoT with ML algorithms. This provides the things in this system with such intelligence that can sense, understand, and act according to the information collected through the sensors installed on personal smart phones. The rest of this paper is organized as follows. "Related work" provides some related works. "The proposed model" discusses the proposed work. "Evaluation and results" presents an evaluation of the proposed model and discusses the results. "Conclusion and future work" provides conclusions and future suggested work.

## Related work

Dorgham et al. [9] proposed a modern hybrid evolutionary approach that incorporates Genetic Algorithm (GA) with efficient evolutionary techniques. A Decision Support System (DSS) was implemented to assist hospital personnel in the assignment operation. The authors demonstrated the efficacy of the proposed approach to solve many benchmark instances recorded in the literature relevant to the smart health-care system using a true deep experimental analysis. In addition, their hybrid algorithm outperforms powerful approaches from the literature that have the best-known outcomes.

Zhou et al. [10] proposed HAR model based on Long-Short Term Memory (LSTM) Deep Learning (DL) algorithm for empowering the Internet of Healthcare Things (IoHT). It used deep Q-network for automatic labeling of data with reward-based on the distance to handle the issue of lack of labeled data. Then, the fusion of user’s body sensors data and environmental data were applied for feeding the model. The results showed that this work outperformed other approaches like SVM, DNN, and Random Forest (RF) with a ROC curve up to 0.95.

Anguita et al. [11] proposed a system based on Support Vector Machines (SVM). The data is collected using a smartphone (Samsung Galaxy S2). Each person of the participants is doing a different activity: laying, walking, sitting, standing, walking up-stairs, and walking down-stairs. The results of the experiments are conducted through comparing two versions of SVM. The performance of the first version, Multi-Class SVM, achieves 89.3% accurate regarding predicting six different classes. However, the second version, Multi-Class Hybrid Fusion SVM, achieves 89% accuracy.

Murad et al. [12] suggested using deep recurrent neural network (DRNN) model. This model helps capturing the entire long-range of relations in the input data rather than being restricted to the size of the kernel window. Also, the model uses three different architectures: unidirectional, bidirectional, and cascading. Performance using DRNN with other algorithms on UCI-HAR dataset is concluded as follows: DRNN has reached the highest accuracy of 96.7% compared to 96% from SVM, and 95.2% from convolution neural network (CNN) and outperformed the others (SVM, K-nearest neighbor, and CNN).

Another proposed an approach for HAR using Deep Belief Neural Network (DBNs) which is built by sequentially stacking multiple Restricted Boltzmann Machines (RBM) [13]. They used a deep activity recognition model with three layers of one thousand neurons each. The results showed that their approach is better than the traditional methods. Also, their results showed that a hybrid DL and Hidden Markov Model (HMM) achieved recognition accuracy of 99.13%.

Chen and Xue [14] presented a CNN model for HAR through modifying the convolution kernel for the purpose of adapting the characteristics of tri-axial acceleration signals. The results showed that their model achieved an accuracy of 93.8% with no using of feature extraction based on a dataset of 31,688 samples gathered from nine activities.

Qin et al. [15] proposed a unique architecture for HAR that utilized data from multiple sensors. This system converts time series data collected from sensors into images. These transformed images were used to keep required features and patterns for the task of HAR. For enabling the model to be trained and evaluated on the collected data from different sensors, the authors used a fusion residual network by merging two networks and training different data pixel-wise correlations. This model provided state-of-the-art performance with an accuracy of 93.41% on HHAR dataset and 98.5% on MHEALTH dataset.

Xia et al. [16] proposed a deep learning model that fuses LSTM layers with convolution layers to draw out the activity attributes without human interference in the feature selection process and classify them correctly. This model collected smartphone sensor data and fed it to two-layer LSTM followed by the convolution layers. The evaluation of the model was carried out on three public datasets. It achieved an accuracy of 95.85%, 95.78%, and 92.63% on WISDM UCI-Har, and OPPORTUNITY datasets, respectively.

Irvine et al. [17] proposed data driven HAR classifier as an ensemble of neural networks (NNs) for improving the quality of the public datasets. They used an ensemble of four NNs which generated and integrated using support function fusion. They introduced different approaches for handling the disputes between the different models. The final ensemble model achieved the best performance that reached an accuracy of 80.39%.

Mliki et al. [18] proposed an approach to HAR using non-invasive means depending on UAV-taken video sequence of human movement. This approach consists of two stages. The first is an offline stage that generates two CNN models (i.e., human/ non-human and the human activity model). The second is the inference stage that is concerned with indicating humans and their activities by adapting CNN. This system outperformed other methods on UCF-ARG dataset with an accuracy of 56% using instance classification and 68% on the entire sequence of frame classification.

Soleimani et al. [19] proposed a new method called Subject Adaptor Generative Adversial Network (SA-GAN). This method helps in handling the issue of the lack of big enough labeled data. The proposed model used GAN framework to execute cross-subject transfer learning in the domain of HAR depending on the data collected from wearable devices. In more than 66% of experiments, the model outperformed other compared approaches, while in the remaining 25% of experiments, it came in second. This work reached of 90% of the accuracy by supervised training over the same domain data in some cases.

Mazzia et al. [20] presented a modified version of capsule networks by substituting the dynamic routing with a novel non-iterative, highly parallelizable routing algorithm that can handle a smaller number of capsules with ease. Extensive testing with other capsule implementations has shown the efficacy of their approach and the potential of capsule networks to effectively embed more generalizable visual representations.

Jiang et al. [21] used artificial neural network (ANN) to approximate the time-dependent distributions of non-Markovian models using solutions of much simpler time-inhomogeneous Markovian models; the approximation does not increase the model's dimensionality while also allowing the kinetic parameters to be inferred. This network is trained using a small number of noisy measurements derived from experimental data or stochastic simulations of the non-Markovian model. They showed that the Markovian models learned by the NN is accurately reflecting the stochastic dynamics across parameter space using a range of models where the delays are caused by transcriptional processes and feedback control.

Attal et al. [22] applied and compared some ML approaches: k-Nearest Neighbor (kNN), SVM, Gaussian Mixture Models (GMM), RF, k-Means, Gaussian mixture models (GMM), k-Means, Gaussian mixture models (GMM), and HMM for HAR. The dataset contains some main daily living human activities. Some of these activities are walking, lying, and standing. They used three inertial wearable accelerometers placement on the human body dataset. Raw data and extracted/selected features were input for the classifiers. The results showed that that KNN has the high performance among all compared approaches. Also, they showed that MM has better performance among the compared unsupervised classifiers.

Shoaib et al. [23] collected data from 13 human activities performed indoors. In these experiments, each participant had a mobile phone in his right pocket and another at his right wrist. Three motion sensors at the wrist and pocket positions based on different scenarios were evaluated. The authors extracted different features for these sensors over different window sizes without overlap. They used Scikit-learn toolkit for analyzing the performance. Naive Bayes (NB), KNN, and decision tree were applied for practical simple and complex activity recognition. Also, they used ten-fold stratified cross-validation. Results proved that there is relatively smaller enhanced recognition because of data combination taken through different sensors at pocket and wrist positions. Also, they showed that increasing size of the window leads to improve the recognition results of various complex activities. However, this factor has a limited effect on the simple activities.

Garcia et al. [24] presented an ensemble called EkVN for HAR. It combines kNN, Decision Tree, and NB. It is based on heuristic hand-crafted feature extraction. The features were extracted from accelerometer, magnetometer, and gyroscope sensors. The results showed the accuracy of EkVN is more sensitive to data from different users to the window size and to the overlapping factor. Also, they [25] presented a multi-classification approach called EAE for HAR using an ensemble of Auto-Encoders (AEs). In EAE, each AE is trained with data for unique class for reconstructing the sensor measurements; each AE is associated with a label/activity. EAE can be updated with the user’s data when loss drops are occurred below a known value. The results of experimentations based on WISDM, MHealth, and PAMAP2 HAR datasets showed that EAE is efficient and competitive among all compared works. Also, they showed that structure of this modular classifier can permit for more flexible models.

Dua et al. [26] developed a DNN-based model that uses CNN, as well as a Gated Recurrent Unit as an end-to-end model that performs automatic feature extraction and activities classification. The raw data is utilized from wearable sensors without using neither pre-processing nor customized features extraction. This work achieved 96.20%, 97.21%, and 95.27%, respectively, on UCI-HAR, WISDM, and PAMAP2 datasets. Overall, the results showed that the performance of the suggested model outperformed other compared works.

Rashid et al. [27] proposed a low-power edge device-friendly Adaptive CNN for energy-efficient HAR called AHAR. During the inference phase, AHAR employs an adaptive design that choices a component of the baseline design to use. Two datasets, Opportunity and w-HAR, were used to validate the work for categorizing locomotor activities. This work achieved a weighted F1 score of 91.79% and 91.57%, respectively, when compared to fog/cloud computing techniques for the first dataset. Also, it achieves F1 score of 97.55% and 97.64%, respectively, on the w-HAR dataset. When compared to the works on the both datasets, this work is much more energy-efficient (422.38 × less) and memory-efficient (14.29 × less).

Mekruksavanich et al. [28] proposed a revolutionary hybrid model called CNN-LSTM to handle HAR problem. It is a deep learning multichannel architecture. Using DHA public dataset of smart-watch accelerometer, the results proved that this model exceeds other compared deep learning approaches in terms of different performance measures. It achieved 96.87% accuracy.

For the HAR challenge, Athavale et al. [29] presented a pre-trained VGG16 model. This CNN model is used to learn the deep features. The signal classification of human activity, which is recorded by the accelerometer sensor of the mobile phone, was done using VGG16. The accelerometer sensor on a smartphone records these data. The features were trained using VGG16 CNN model is fifth max-pooling layer and fed to SVM. The fully connected layer of this model was replaced by the SVM classifier. This work achieved 79.55% accuracy and 71.63% F-Score based on UniMiB dataset that includes samples of human everyday life activity.

Shang et al. [30] proposed a WiFi-based HAR system. This system can determine different activities via the Channel State Information (CSI) from WiFi devices. They presented a special deep learning framework, LSTM-CNN. It can automatically extract features from temporal and spatial domains. The authors proved the effectively of their work in classifying different activities. Also, the experimentations results proved that this work is better than the compared models on HAR of CSI data; it achieves an average accuracy of 94.14% in multi-activity classification.

Poma et al. [31] presents a way to search for the best number of filters for each convolution layer of a CNN. They advocated searching for the best number of filters in the convolution layer of CNN. In addition, to identify the parameters of the fuzzy system memberships, they applied Fuzzy Gravitational Search Algorithm approach. ORL dataset is used that contains 40 images of different human faces with ten images for each face. The results proved that this work achieves a high%age of recognition.

## The proposed model

**Fig. 1:**
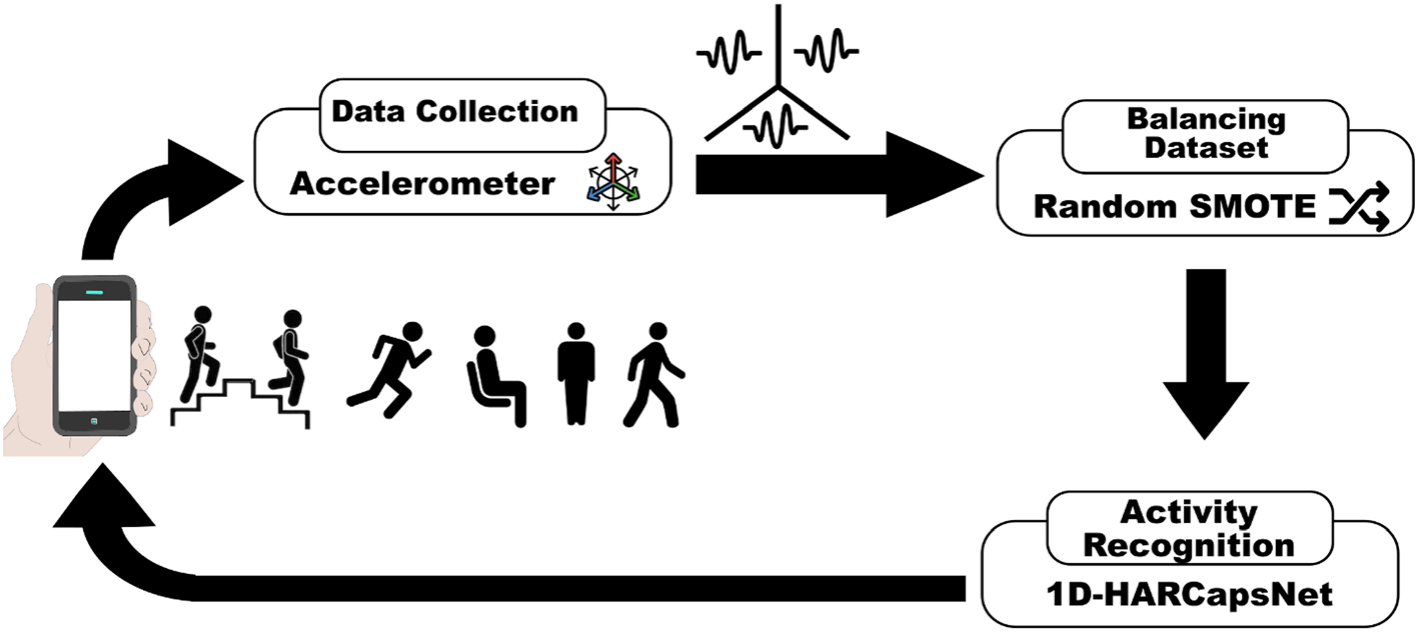
The overall proposed framework

This paper proposes an intelligent decision support system for recognizing the human’ daily activities that feed the sensing data to the recognition model after handling their imbalanced issues. Figure 1 show our overall proposed framework. It has three steps:

- **Data collection** Tri-axial accelerometers which are integrated in the smartphone have been used for gathering 3D time-series data that represent the linear acceleration based on vibration in three directions *X*, *Y* and *Z*. Our model uses the raw Wireless Sensor Data Mining (WISDM) dataset [32].
- **Balancing dataset** This is done by applying the random oversampling technique to handle the issues of biased dataset.
- **Activity recognition** A modified version of 1-D capsule neural network was used to recognize the activities which were exercised and notify the user with the activity class in accordance with the sensor’s readings.

### Using over-sampling for balancing the dataset

In WISDM dataset [32], the samples that represent walking and jogging activity classes out-number the samples of the other classes by large margin. Due to the imbalanced behavior of WISDM dataset that adversely affect the performance of the classifier, the Random-SMOTE algorithm [33] is used to increase the number of the minority class to reach the optimal balanced ratio of 1:1. This is done by randomly selecting examples from the minority class and adding them to the training dataset. For a dataset that has N attributes, taking an attribute n as a sample, the new value is randomly generated using the Random-SMOTE algorithm [33].

### Proposed 1D capsule neural network for HAR

**Fig. 2:**
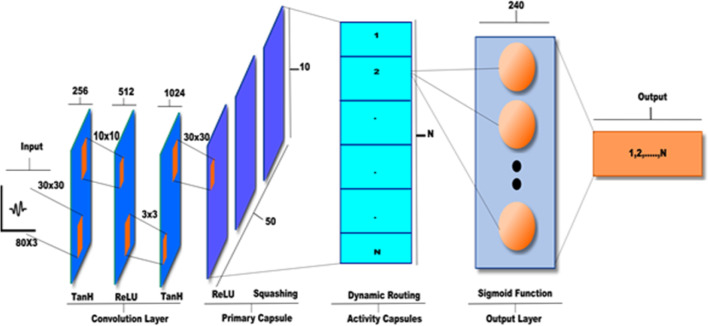
Structure of proposed 1D-HARCapsNet

A capsule neural network (CapsNet) is a newly developed machine learning that was introduced in [34] as a development of CNN. The idea behind its architecture came from adding structures known as “capsules” to a CNN. Capsules are structures of neurons that are activated when a set of attributes are related to a class activity. Usually, an artificial neuron produces a single value and formally a scalar value is related to the probability of the existence of the class in the feature vector. In CapsNet, the scalar output is replaced with the vector-based capsules. The output of the higher capsule (parent) is computed by the scalar product of the coefficient representation of the probabilities of its related lower capsules (children). The closer the child to the parent is, the higher the coefficient between the parent and the child is. In this paper, we propose 1D-HARCapsNet model as a modified version of 1D capsule neural network presented by Suri and Gupta [35]. The proposed model is applied for recognizing the human activities based on the immediate observations of the human actions. Instead of using a single level of convolutional layer, 1D-HARCapsNet architecture implements three levels 1- D convolutional layer (3-Conv1D). The rest of the architecture comprises the primary capsule layer, the activity capsule layer, and the output layer. Figure 2 shows the structure of the proposed 1D-HARCapsNet from the input to the output.

The input data has 80 3D vectors (80 × 3). The model feeds the data to three consequent levels of convolution layer (3-Conv1D) of sizes (80 × 3, 51 × 256 and 42 × 512) respectively. Next, it uses the primary capsule convolution layer of size 40 × 1024 where its generative output is sent to the fully connected activity layer that produces a scalar vector. Finally, this value is passed to the output layer which generates the most likely target class. Table 1 illustrates the structure of the proposed 1D-HARCapsNet model.

**Table 1:** Structure of proposed 1D-HARCapsNet

Three level convolution (3-Conv1D)			Capsule layer	Activity layer	Output layer
1st level	2nd level	3rd level	(1-Conv1D) layer		
80 × 3	51 × 256	42 × 512	40 × 1024	6 × 10	1 × 1

#### The 3-Conv1D layer

Input data samples with (80 × 3) size represent 80 data point wide with the height of three data points are fed into a sequence of three Conv1D with different activation functions to construct the feature maps. The first level of the 3-Conv1D implements 256 filters with a kernel size of (30 × 30) and uses the tanh activation function to calculate the hyperbolic tangent value of the given input. The output is 51 data points wide and the height is 256 data points which is sent to the next level. The Second level implements 512 filters with a kernel size (10 × 10) and uses the ReLu activation function that generates the input directly if it is not negative, otherwise it will output zero. The output of this level is 42 data points wide and 512 data point height which is sent to the last level of the 3-Conv1D layer. The third level implements 1024 filters with a kernel size (3 × 3) and uses the tanh activation function. Totally, the output of this layer is 40 data points wide and 1024 height data points which is sent to the next layer as an array of feature maps for further processing.

#### The primary capsule layer

The primary capsule layer is a 1-D convolution (Conv1D) layer with a kernel size (30 × 30). It implements the reshape function to convert the array of the feature maps into the corresponding vectors. Finally, it is passed to the squashing function to convert the vector output to a value between 0 and 1.

#### The activity capsule layer

It replaces each capsule in the network with its actual class activity by implementing the dynamic routing algorithm. Routing by agreement is based on the ability of the lower capsule (*i*) in the primary capsule layer to predict the output of the higher capsule (*j*) in the activity capsule layer.

For each capsule *i* and capsule *j*, the prediction of the output of capsule *j* is denoted by *U*_*j*|*i*_ and calculated by Eq. 1:

1 $$ U_{j|i} = W_{ij} u_{i} , $$

where *u*_*i*_ represents the output of the capsule *i* and *W*_*ij*_ is the weight matrix. Next, the total input *S*_*i*_ to capsule *j* in the activity capsule layer is calculated using a weighted sum overall the prediction vectors as given in Eq. 2.

2 $$ S_{i} = \sum_{i} C_{ij} U_{j|i} , $$

where *C*_*ij*_ are the coupling coefficients between the capsule *i* and all the capsules in the higher layer. It is calculated using a routing softmax function as given in Eq. 3.

3 $$ c_{ij} = { }\frac{{{\text{exp}}\left( {b_{ij} } \right)}}{{\mathop \sum \nolimits_{k} {\text{exp}}\left( {b_{ik} } \right)}}, $$

where *b*_*ij*_ indicates log prior probability of the capsule *j* in coupled to capsule *i*, *k*. Finally, the scalar output vector of capsule *j* is obtained by applying a non-linear squashing function to its total input according to Eq. 4.

4 $$ v_{j} = \frac{{\left| {\left| {S_{j} } \right|} \right|^{{2{ }}} }}{{1 + \left| {\left| {S_{j} } \right|} \right|^{{2{ }}} }}\frac{{S_{j} }}{{\left| {\left| {S_{j} } \right|} \right|}}. $$

#### The output layer

The output layer is a fully connected layer that consists of 240 sigmoid units that predicts the most likely target class activity *y* based on the scalar vector *x *as illustrated in Eq. 5.

5 $$ y = \frac{1}{{1 + e^{ - x} }}. $$

## Evaluation and results

In the evaluation process, the widely used criteria such as: accuracy, precision, recall, and F-measure will be used. All the four criteria depend on the confusion matrix [36].

### Evaluation criteria

Multiple performance evaluation criteria are used for ensuring the improvement of the proposed model compared to other existing models. The confusion matrix [36] is one of the most used evaluation metrics in the field of machine learning. Correct predication is considered as True Positive (*TP*), but if it is negative and is predicted as such, it is considered True Negative (*TN*). If it is negative and classified as positive, this is considered False Positive (*FP*). In case it is positive and classified as negative, this is considered False-Negative (*FN*). The confusion matrix values are used for measuring other important metrics such as: geometric mean, accuracy, error rate, recall, and F1-measures). Accuracy [37] is the correctly predicted samples rate. It is the ratio between correctly predicted samples to the total number of samples due to its straightforward meaning. It is one of the most used metrics in the field of the machine learning evaluation as illustrated in Eq. 6:

6 $$ {\text{Acc}} = \frac{{{\text{TP}} + {\text{TN}}}}{{{\text{TP}} + {\text{Tn}} + {\text{FP}} + {\text{FN}}}}. $$

Precision (positive predictive) [37] is the ratio of correctly predicted positive class to the total number of the positive predicted samples in the dataset as illustrated in Eq. 7:

7 $$ {\text{PPV}}\;\left( {{\text{Precision}}} \right) = \frac{{{\text{TP}}}}{{{\text{FP}} + {\text{TP}}}}. $$

Recall or hit rate or true positive rate (TPR) is also known as sensitivity such as in [37]. It is the rate of corrected predicted samples to the total number of positive samples in the dataset as illustrated in Eq. 8:

8 $$ {\text{Recall}}\;\left( {{\text{TPR}}} \right) = \frac{{{\text{TP}}}}{{{\text{TP}} + {\text{FN}}}}. $$

F1-measure [37] is also called F-measure. It presents the harmonic means between precision and recall as illustrated in Eq. 9:

9 $$ F1{\text{-measure}} = \frac{{2 \times {\text{Precision}}\;\left( {{\text{PPV}}} \right) \times {\text{Recall}}\;\left( {{\text{TPR}}} \right)}}{{{\text{Precision}}\;\left( {{\text{PPV}}} \right) + {\text{Recall}}\;\left( {{\text{TPR}}} \right)}}. $$

### Wireless sensor data mining (WISDM) dataset

WISDM time-series dataset is used for the task of (HAR) using the tri-axial accelerometer sensor on most android smartphones [32]. It consists of 1,098,207 different examples and each one consists of six different attributes with class distribution [walking: 424,400 (38.6%), jogging: 342,177 (31.2%), upstairs: 122,869 (11.2%), downstairs: 100,427 (9.1%), sitting: 59,939 (5.5%), standing: 48,395 (4.4%)] as illustrated in Table 2.

**Table 2:** Raw examples distribution

Walking	Jogging	Upstairs	Downstairs	Sitting	Standing
38.6%	31.2%	11.2%	9.1%	5.5%	4.4%

### The hyper parameters of the proposed 1D-HARCapsNet

This paper introduces 1DHARCapsNet model with the following hyper parameters. The number of epochs is 25 and 50, the learning rate values are 0.001 and 0.002, the number of routing are5 and 10, and the initial weights are 0.002, 0.003, 0.004 and 0.005 as illustrated in Table 3.

**Table 3:** The hyper parameters of the proposed 1D-HARCapsNet

Epochs	Learning rate	Routing	Weights
25, 50	0.001, 0.002	5, 10	0.002, 0.003, 0.004, 0.005

### Recognition experiments of the proposed 1D-HARCapsNet

We have conducted our experiments on Kaggle cloud in which the dataset was split into 80% for training and 20% for testing. Table 4 shows the used hardware specifications.

**Table 4:** Experiments hardware specifications

Graphical processing unit (GPU)	Central processing unit (CPU)	Hard disk	Operating system
NVIDIA Tesla P100—16 GB Ram	Single core Intel Xeon CPU -2.3 GHz	73 GB	Linux-SMP Debian

**Fig. 3:**
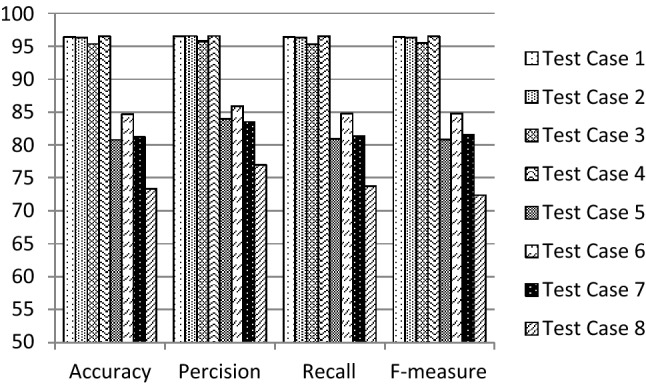
Evaluation results of the suggested test cases (1–8) for 1D-HARCapsNet

**Fig. 4:**
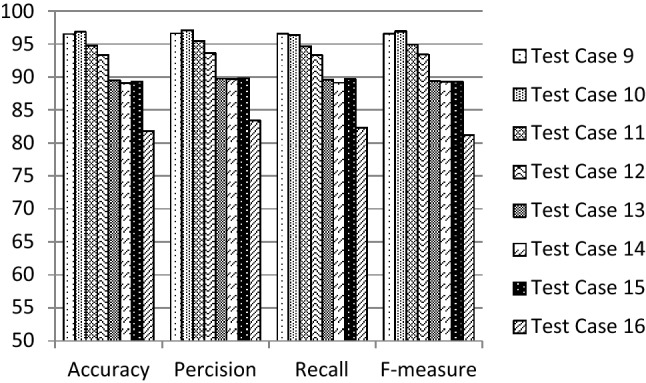
Evaluation results of the suggested test cases (9–16) for 1D-HARCapsNet

**Fig. 5:**
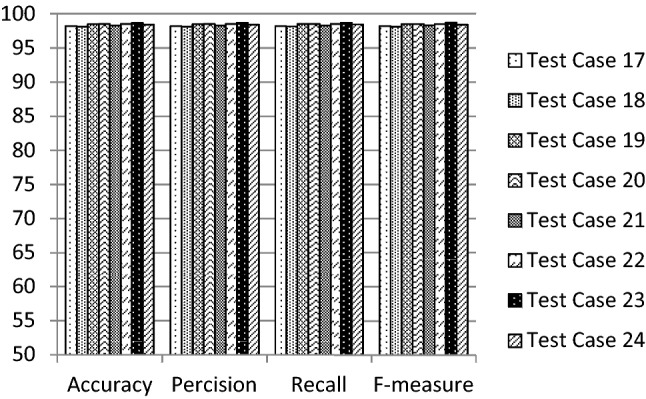
Evaluation results of the suggested test cases (17–24) for 1D-HARCapsNet

**Fig. 6:**
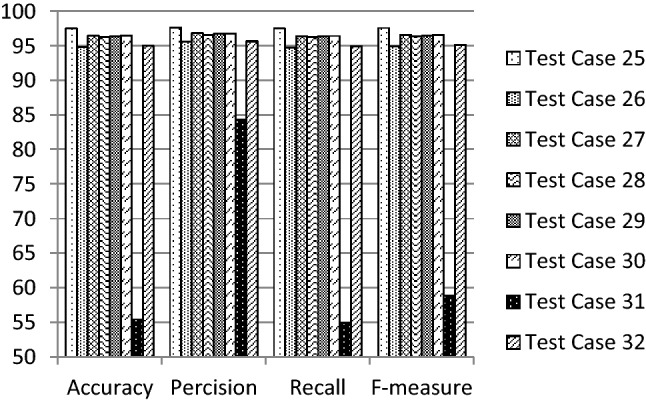
Evaluation results of the suggested test cases (25–32) for 1D-HARCapsNet

The performance of the proposed 1D-HARCapsNet model is compared with the conventional one-dimensional deep capsule network architecture [35] having the same hyper parameters indicated in Table 3. A series of experiments were conducted to evaluate the results by constructing different 32 test cases generated using the suggested hyper parameters. Table 5 illustrates the variation of the conventional CapsNet recognition results. The best achieved results are 90.11% accuracy, 91.81% precision, 89.94% recall and 0.903F-measure. Table 5 results on the WISDM dataset using the modified architecture without applying Random SMOTE. Table 7 illustrates the variation of the proposed system recognition results. In this model the data is balanced using random SMOTE algorithm and then it is being fed to the proposed structure of 1D-HARCapsNet with the above-mentioned hyper parameters. Figures 3, 4, 5 and 6 show the evaluation results of the constructed test cases. The accuracy values varied from 73.39 to 98.67%, the precision values varied from 76.97 to 98.66%, the recall values varied from 73.77 to 98.67% and the F-measure values varied from 0.724 to 0.987. The best recognition results achieved are based on using the values of 25, 0.002, 10 and 0.002 for the number of epochs, learning rate, routing, and weights, respectively (Tables 6, 7).

**Table 5:** Recognition results of conventional CapsNet model [35]

Hyper parameters				Recognition results			
#Epoch	Learning rate	#Routing	Weights	Accuracy (%)	Precision (%)	Recall (%)	F-measure
25	0.001	5	0.004	87.91	90.52	87.92	0.882
		5	0.005	88.70	91.46	88.88	0.891
		10	0.004	67.43	86.53	67.71	0.709
		10	0.005	67.97	86.79	68.29	0.715
	0.002	5	0.004	69.70	84.75	69.87	0.724
		5	0.005	78.41	82.16	78.41	0.786
		10	0.004	66.95	81.69	67.07	0.693
		10	0.005	69.15	82.1	69.24	0.716
50	0.001	5	0.004	90.11	91.81	89.94	0.903
		5	0.005	70.41	86.97	70.65	0.735
		10	0.004	65.31	82.05	65.45	0.679
		10	0.005	67.43	82.73	67.52	0.702
	0.002	5	0.004	74.73	80.69	74.94	0.747
		5	0.005	70.72	80.69	71.05	0.716
		10	0.004	16.33	2.72	16.67	0.047
		10	0.005	74.1	84.89	74.78	0.728
25	0.001	5	0.002	75.12	88.26	75.44	0.778
		5	0.003	76.37	87.98	76.39	0.780
		10	0.002	69.31	87.28	69.56	0.727
		10	0.003	67.03	86.02	67.21	0.705
	0.002	5	0.002	83.59	89.54	83.26	0.838
		5	0.003	70.8	87.1	71.04	0.739
		10	0.002	73.39	82.39	73.35	0.745
		10	0.003	64.13	80.97	64.3	0.668
50	0.001	5	0.002	71.51	86.47	71.86	0.744
		5	0.003	86.73	90.29	86.78	0.873
		10	0.002	67.43	83.3	67.48	0.7
		10	0.003	72.68	86.46	72.86	0.753
	0.002	5	0.002	76.06	87.71	76.67	0.757
		5	0.003	70.96	84.64	71.55	0.698
		10	0.002	17.82	2.97	16.67	0.05
		10	0.003	75.98	81.88	76.18	0.765

**Table 6:** Results of a modified architecture without applying random-SMOTE algorithm on the WISDM dataset

Hyper parameters				Recognition results			
#Epoch	Learning rate	#Routing	Weights	Accuracy (%)	Precision (%)	Recall (%)	F-measure
25	0.001	5	0.004	95.09	94.5	91.75	0.930
		5	0.005	96.0	95.0	93.33	0.941
		10	0.004	96.55	95.62	93.91	0.947
		10	0.005	96.73	96.45	94.85	0.956
	0.002	5	0.004	78.91	82.31	70.26	0.736
		5	0.005	81.82	88.59	70.91	0.76
		10	0.004	79.82	73.7	74.46	0.682
		10	0.005	81.45	74.71	75.75	0.706
50	0.001	5	0.004	90.18	91.82	85.47	0.875
		5	0.005	89.09	93.56	93.56	0.896
		10	0.004	94.18	94.7	91.5	0.929
		10	0.005	94.18	95.15	91.38	0.93
	0.002	5	0.004	86.0	78.07	77.92	0.732
		5	0.005	82.91	77.02	75.8	0.716
		10	0.004	81.64	78.59	72.93	0.69
		10	0.005	71.09	64.83	60.11	0.532
25	0.001	5	0.002	95.82	94.03	93.85	0.939
		5	0.003	95.82	94.19	93.82	0.94
		10	0.002	96.0	95.07	94.38	0.947
		10	0.003	96.0	94.19	93.56	0.939
	0.002	5	0.002	75.27	78.76	61.21	0.646
		5	0.003	80.18	81.53	73.31	0.753
		10	0.002	77.45	76.2	69.37	0.646
		10	0.003	77.45	75.29	69.7	0.641
50	0.001	5	0.002	86.18	91.22	77.17	0.823
		5	0.003	87.09	91.82	82.84	0.862
		10	0.002	91.64	89.98	86.43	0.875
		10	0.003	93.82	94.78	90.93	0.926
	0.002	5	0.002	88.55	81.83	83.44	0.815
		5	0.003	83.64	88.44	73.15	0.762
		10	0.002	84.73	83.78	78.31	0.796
		10	0.003	89.64	88.27	84.65	0.862

**Table 7:** Results of 1D-HARCapsNet based on the hyper parameters

Hyper parameters				Test cases	Recognition results			
#Epoch	Learning rate	#Routing	Weights	T1	Accuracy (%)	Precision (%)	Recall (%)	F-measure
25	0.001	5	0.004	T2	96.39	96.52	96.39	0.964
		5	0.005	T3	96.31	96.54	96.26	0.963
		10	0.004	T4	95.37	95.77	95.34	0.955
		10	0.005	T5	96.47	96.55	96.5	0.965
	0.002	5	0.004	T6	80.77	84.0	80.9	0.808
		5	0.005	T7	84.69	85.9	84.75	0.848
		10	0.004	T8	81.24	83.5	81.38	0.815
		10	0.005	T9	73.39	76.97	73.77	0.724
50	0.001	5	0.004	T10	96.55	96.63	96.57	0.966
		5	0.005	T11	96.86	97.06	96.83	0.969
		10	0.004	T12	94.74	95.49	94.68	0.949
		10	0.005	T13	93.33	93.66	93.37	0.934
	0.002	5	0.004	T14	89.48	89.72	89.59	0.894
		5	0.005	T15	89.09	89.66	89.13	0.893
		10	0.004	T16	89.32	89.83	89.65	0.893
		10	0.005	T17	81.79	83.38	82.29	0.812
25	0.001	5	0.002	T18	98.19	98.21	98.18	0.982
		5	0.003	T19	98.12	98.12	98.15	0.981
		10	0.002	T20	98.50	98.49	98.52	0.985
		10	0.003	T21	98.51	98.51	98.52	0.985
	0.002	5	0.002	T22	98.27	98.27	98.28	0.983
		5	0.003	T23	98.51	98.52	98.53	0.985
		10	0.002	T24	98.67	98.66	98.67	0.987
		10	0.003	T25	98.43	98.43	98.44	0.984
50	0.001	5	0.002	T26	97.49	97.59	97.45	0.975
		5	0.003	T27	94.82	95.54	94.74	0.949
		10	0.002	T28	96.39	96.77	96.33	0.965
		10	0.003	T29	96.23	96.51	96.21	0.963
	0.002	5	0.002	T30	96.31	96.66	96.27	0.964
		5	0.003	T31	96.39	96.7	96.35	0.965
		10	0.002	T32	55.42	84.27	54.92	0.589
		10	0.003	T33	94.98	95.6	94.9	0.951

### Comparing the proposed model against other models

Table 8 illustrates the obtained accuracy, precision, recall and F-measure of our proposed model compared with the state-of-the-art models [38–45] on raw version of WISDM dataset. The Accuracy of the proposed model has the highest accuracy of 98.67%. In the second place, Spatio-Temporal Deep Learning [46] has accuracy of 98.53%, in third-place Deep learning low power device [41] has accuracy of 98.2% while in the third-place, CNN + BLSTM [44] has accuracy of 97.8%. Based on Precision, the proposed model has achieved the highest precision of 98.66%. In the second place, Random Forest Classifier [43] has precision of 98.1% while in the third-place CNN + BLSTM [44] has precision of 97.8%. Based on recall, the proposed model has achieved the highest recall of 98.67%. In the second place, Random Forest Classifier [43] has recall of 98.1% while in the third-place, CNN + BLSTM) [44] has recall of 97.8%. On basis F-measure, the proposed model has achieved the highest F-measure with 0.987. In the second the place, the Random Forest Classifier [43] has 0.981of F-measure while in the third place, CNN + BLSTM [44] has 0.978 of F-measure. Generally, the proposed model has performed the best across the four performance evaluation criteria.

**Table 8:** A comprehensive comparison of multiple methods on WISDM dataset

Reference	Method	Accuracy (%)	Precision (%)	Recall (%)	F-measure (%)
[45]	Handcrafted features + Dropout	85.36	N/A	N/A	N/A
	CNN + stat. features + interval size 50	90.42	N/A	N/A	N/A
	CNN + stat. features + interval of size 200	93.32	N/A	N/A	N/A
	Basic Features + RF + interval of size 200	82.66	N/A	N/A	N/A
[38]	U-Net	97	N/A	N/A	0.970
	FCN	86.2	N/A	N/A	0.861
	CNN	95.8	N/A	N/A	0.958
[39]	Ensemble classifiers + 10 s window	94:3	N/A	N/A	N/A
[40]	Deep learning low power device + 10 s window	98.2	N/A	N/A	N/A
[41]	RNN	81.74	N/A	N/A	N/A
	CNN	92.22	N/A	N/A	N/A
	KNN + K-fold cross validation	90.19	N/A	N/A	N/A
[42]	Random Forest Classifier	98.09	98.1	98.1	0.981
[43]	J-RIP	N/A	94.3	94.3	0.942
[44]	CNN + BLSTM	97.8	97.8	97.8	0.978
[47]	Hybrid deep learning approaches	97.77	N/A	N/A	N/A
[46]	Spatio-Temporal Deep Learning	98.53	N/A	N/A	N/A
[48]	optimization of Deep Learning using Genetic Algorithm	94.5	N/A	N/A	N/A
[26]	Multi-input CNN-GRU	97.21	N/A	N/A	97.22
[49]	LSTM	N/A	9704	9704	0.974
[35]	Conventional CapsNet	90.11	91.81	89.94	0.903
	Proposed 1D-HARCapsNet	98.67	98.66	98.67	0.987

## Conclusion and future work

In this paper, a modified version of the 1-D capsule neural network called 1DHARCapsNet was proposed to provide an efficient intelligent decision support approach for recognizing the human activity. We implemented the Random SMOTE algorithm to handle the issue of imbalanced behavior of WISD dataset. The proposed model comprises four layers: 3-Conv1D layer, the primary capsule layer, the activity capsule layer, and the output layer. The experimental results were evaluated on a raw version of WISDM dataset. The performance was assessed based on the four criteria: accuracy, precision, recall, and F-measure. Compared to the state-of-the-art algorithms, the proposed model proved its ability to recognize the human activity and outperform the others.

In the future studies, we suggest using Gray Wolf Optimizer (GWO) [50] for feature selection to improve the performance to surpass the-state-of-the-art algorithms and to provide optimal performance. GWO helps reducing the effects of noise and redundancy of data on the overall performance of the system, especially accuracy. Also, in the future work, optimization of the proposed model for different embedded devices will be performed to embed the classifier within power constrained microcontrollers, and to ensure the security of user’s data and preserve its privacy.
